# Modified Zenker's peroral endoscopic myotomy for giant Zenkerʼs diverticulum using a tunnel-free approach, undersaline immersion and mucosal trimming

**DOI:** 10.1055/a-2835-1647

**Published:** 2026-04-02

**Authors:** Georgios Mavrogenis, Stamatina Vogli, Georgios Kanoupakis

**Affiliations:** 1 168211 Third Space Endoscopy Unit, Mediterraneo Hospital, Athens, Greece

Zenker’s peroral endoscopic myotomy (Z-POEM) has emerged as an effective endoscopic technique for the treatment of Zenker’s diverticulum. Several technical refinements have been proposed, including direct septal mucosal incision 1 , tunnel-free approaches 2 3 , and mucosal flap incision after septotomy 4 . Here, we present a modified Z-POEM technique applied in a patient with a giant Zenker’s diverticulum ( Video 1 ).

Modified Z-POEM for giant Zenker’s diverticulum.Video 1

**Fig. 1:**
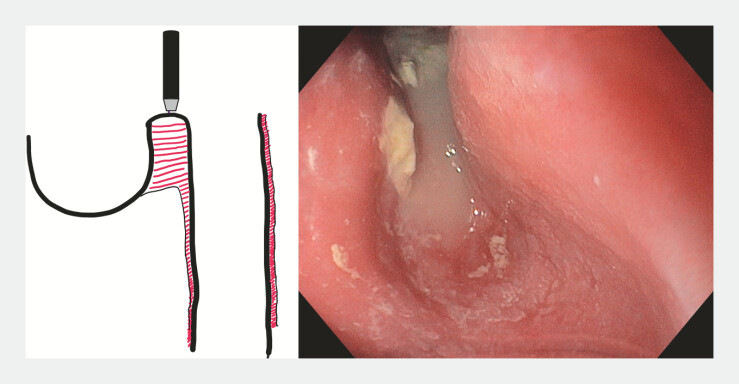
The 9-cm Zenker’s diverticulum.

**Fig. 2:**
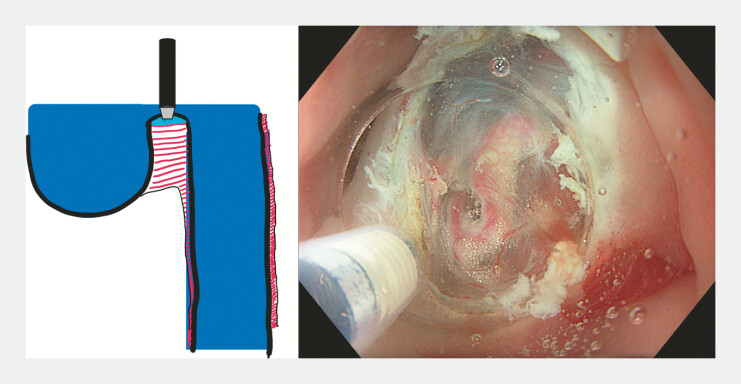
Under-saline mucosotomy over the septum.

**Fig. 3:**
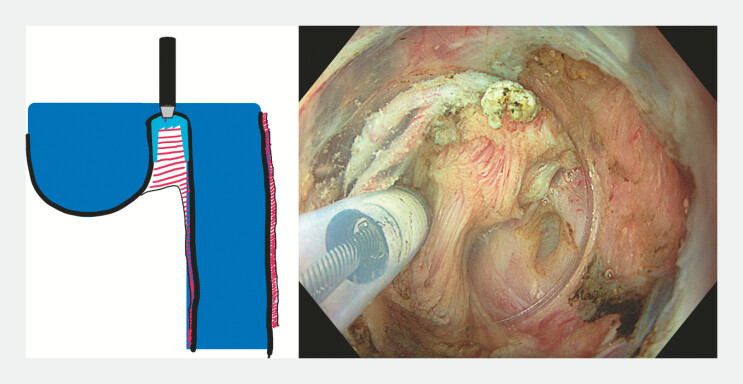
Under-saline tunnel-free myotomy.

A 59-year-old patient presented with severe dysphagia caused by a 9-cm Zenker’s diverticulum. Endoscopy confirmed the large pouch and an associated tight upper esophageal stricture. After multidisciplinary discussion, modified Z-POEM was performed incorporating several adaptations. Myotomy was initiated directly over the septum to facilitate access ( Fig. 1 ) with Flush Knife BTs 1.5 (Fujifilm, Tokyo, Japan). The lumen was filled with saline, enabling under-saline visualization and safe identification of the submucosal plane ( Fig. 2 ). Generous bilateral submucosal injections created protective cushions allowing direct septotomy despite limited working space ( Fig. 3 ).

**Fig. 4:**
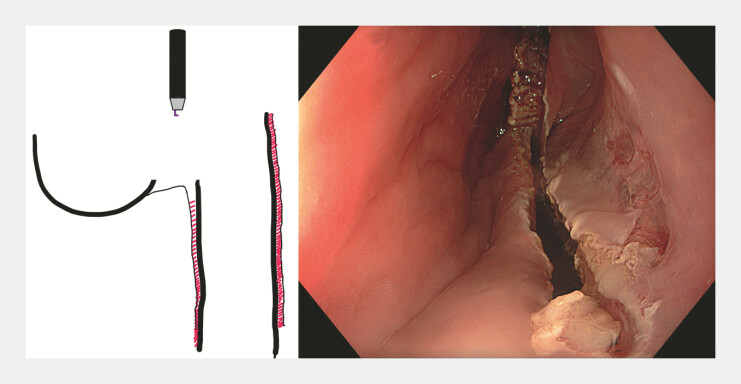
Central incision of the giant flap.

After approximately 2-cm myotomy, exposure improved and dissection continued under CO₂ insufflation. The myotomy was extended to the diverticular base and approximately 2 cm into the esophagus to minimize recurrence risk. Adequate depth was indirectly assessed by mucosal discoloration at the diverticulum base. Because of the broad septal surface, both central and lateral incisions were performed. The oversized mucosal flap was symmetrically trimmed using a Hook knife (Olympus, Tokyo, Japan; Fig. 4 ).

**Fig. 5:**
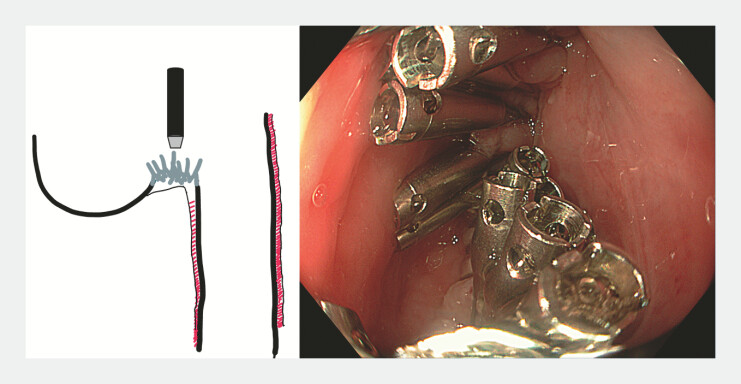
Clip closure in a V-shape fashion.

Closure was achieved with multiple clips ( Fig. 5 ). Postoperative esophagram showed no leakage or retention. Recovery was uneventful. At a 6-month follow-up, the patient remains asymptomatic with normal oral intake.

This modified approach combines under-saline immersion, tunnel-free septotomy, extended esophageal myotomy, and mucosal trimming to optimize safety and efficacy in large Zenker’s diverticula and may reduce the recurrence risk. Further studies are needed to confirm long-term clinical outcomes.

Endoscopy_UCTN_Code_TTT_1AO_2AP
